# In Experimental Dilated Cardiomyopathy Heart Failure and Survival Are Adversely Affected by a Lack of Sexual Interactions

**DOI:** 10.3390/ijms21155450

**Published:** 2020-07-30

**Authors:** Ranjana Tripathi, Ryan D. Sullivan, Tai-Hwang M. Fan, Radhika M. Mehta, Inna P. Gladysheva, Guy L. Reed

**Affiliations:** 1 Department of Internal Medicine, University of Arizona College of Medicine—Phoenix, Phoenix, AZ 85004, USA; rtripathi@arizona.edu (R.T.); ryansullivan@arizona.edu (R.D.S.); rmmehta@arizona.edu (R.M.M.); 2 Department of Medicine, University of Tennessee Health Science Center, Memphis, TN 38163, USA; tfan1@uthsc.edu

**Keywords:** heart failure, dilated cardiomyopathy, edema, pleural effusion, contractile function, testosterone, lifespan

## Abstract

Nearly one in three people in the U.S. will develop heart failure (HF), characterized by fluid retention (edema) in the lungs and elsewhere. This leads to difficult breathing, deterioration of physical capacity, restriction of normal activities and death. There is little data about the safety and effects of sexual interactions in patients with HF. We tested whether a lack of sexual interactions affected pathophysiological outcomes in a pre-clinical mouse model of dilated cardiomyopathy that recapitulates the progressive stages of human HF. Male mice were randomly given access to, or deprived from, sexual interactions with female mice, which were confirmed by videography and generation of offspring. Cohousing with access to sexual interactions markedly prolonged survival, while cohousing without access to sexual activity did not. Sexual interactions improved systolic function, reduced HF-associated edema, altered transcription of heart contractile protein genes and decreased plasma testosterone levels. To determine whether testosterone levels contributed to survival, testosterone levels were experimentally reduced. Reduction of testosterone levels significantly prolonged survival. Taken together, in mice with dilated cardiomyopathy, sexual activity altered cardiac contractile gene transcription, improved systolic function, reduced edema and prolonged survival which may be in part due to lower testosterone levels.

## 1. Introduction

Dilated cardiomyopathy (DCM) is a major cause of severe heart failure (HF), which is characterized by retention of fluids (edema) in the lungs and elsewhere, leading to difficulty with breathing, deterioration of physical capacity, restriction of normal daily activities and death [1,2,3]. For individuals with and without HF, sexual activity enhances the quality of life, which patients often rank as more important than length of life alone [4,5,6]. Sexual activity is associated with moderate physical exertion, which is tolerable in patients with mild HF, but it may be frightening or difficult for those with diminished physical capacity due to severe HF [7]. The safety and long term effects of sexual activity in patients with HF are not fully known due to a paucity of experimental data [5,6]. Health care professionals frequently do not consider sexual activity in their care for HF patients [8,9,10]. Examining the effects of sexual activity in human HF has been challenging as patients are heterogeneous, may have comorbidities and options for experimental intervention are limited.

**Figure 1:**
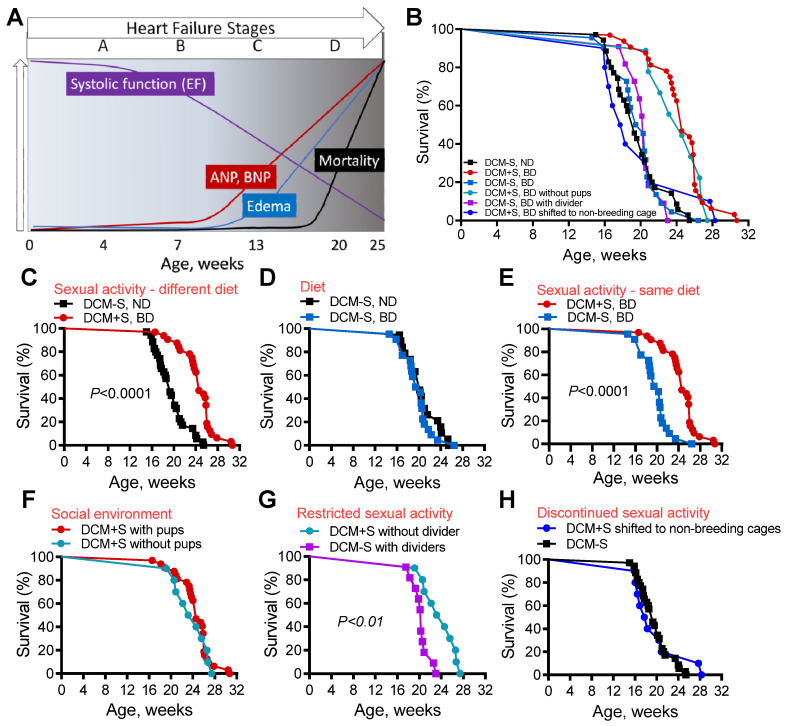
Access to sexual activity improves survival. (**A**) Schema of heart failure stages, biomarkers, systolic function and mortality in male dilated cardiomyopathy (DCM) mice [12,19,21]. Ejection frication (EF); Atrial and B-type natriuretic peptide (ANP and BNP). (**B**) Kaplan–Meier survival curves for DCM mice under all different conditions: DCM mice with (DCM+S) or without access to sexual interactions (DCM-S), with normal diet (ND) or breeding diet (BD), with or without pups in the cage, with or without a cage divider, and with separation and shifting to other cages. (**C**–**H**) Relevant pairwise comparisons of Kaplan-Meier survival curves (from panel B) for DCM mice under different conditions. (**C**) DCM mice with access to sexual interactions with females on a breeding diet (DCM+S, BD, *n* = 32) vs. DCM mice without access on a normal, non-breeding diet (DCM-S, ND, *n* = 35). (**D**) Effect of diet on survival, DCM-S on a normal diet (DCM-S, ND, *n* = 19) vs. DCM-S on breeding diet (DCM-S, BD, *n* = 22). (**E**) DCM mice with or without sexual interactions on the same breeding diet. (**F**) Effect of co-housing with pups: DCM+S cohoused with pups (*n* = 32) vs. DCM+S housed without pups (*n* = 10). (**G**) Effect of co-housing with females without sexual activity: DCM-S (*n* = 10) mice co-housed with female mice with sexual activity restricted by porous cage dividers between male and female mice, see S1B) vs. DCM+S (*n* = 32) in which sexual interactions were not restricted. (**H**) Effect of terminating sexual interactions with females: DCM-S (*n* = 35) without continuous access to sexual interactions with females vs. DCM+S in which sexual access was terminated by transfer to non-breeding cages at 13 weeks of age (*n* = 11). Survival of control mice without DCM was normal and consistent with wild-type C57BL6/J mice.

Patients with HF from DCM suffer from progressive heart enlargement and decline in the pumping or contractile function of the heart [3,11]. DCM has genetic and other causes [3,11], but it typically progresses to symptomatic HF through four stages (A–D) [12], which are recapitulated in a well-characterized mouse model of DCM-HF with reduced ejection fraction (Figure 1A) [13,14,15,16,17,18,19,20,21]. Stage A HF occurs in those with genetic or other risk factors for DCM and Stage B marks the initial occurrence of heart disease. Stage C begins with the development of symptomatic edema HF and Stage D signifies severe end-stage HF [12,22]. Similar to humans, mice with DCM pass through all of these stages (Figure 1A), from normal health (Stage A) to progressively declining contractile function, increasing heart dilation (Stage B), to the development of edema or fluid retention (Stage C) with increases in associated biomarkers (atrial natriuretic peptide, ANP and B-type natriuretic peptide, BNP, etc.), to the onset of severe debilitating HF (Stage D) with left atrial appendage thrombus formation (LAAT) and death [13,19,21]. Mice with DCM are responsive to treatments with survival benefits [16,18,20]. Thus, this DCM-HF model is compliant with the requirements of the American Heart Association Scientific Statement for preclinical animal models of HF [22].

In this study we examined the effect of deprivation from sexual activity on pathophysiological outcomes in translationally relevant mouse model of DCM-HF that recapitulates the progressive stages of human HF [13,14,15,16,17,18,19,20,21]. We used human clinical diagnostic modalities (echocardiography and magnetic resonance imaging) to monitor progression of HF [23]. We show that access to sexual interaction improves heart function, slows HF development, alters cardiac contractile gene expression and prolongs life in mice with DCM.

## 2. Results and Discussion

### 2.1. Access to Sexual Activity Improves Survival in Mice with Dilated Cardiomyopathy (DCM)

To propagate DCM mice, male DCM mice were bred with congenic, female non-transgenic without DCM, because similar to humans, pregnancy in female DCM mice is associated with significant cardiovascular stress and fatality [24]. As we monitored these mice, we found that male DCM mice with sexual interactions (DCM+S) due to co-housing with female mice lived significantly longer (Figure S1A) than DCM mice that were co-housed with male mice and had no access to female sexual interactions (DCM-S).

To rigorously test whether access to sex affected survival, male DCM littermate mice at Stage B HF (6–7 weeks old) were randomly assigned to domiciling or co-housing with congenic females (DCM+S) or with male littermates (DCM-S). Mice were housed under identical conditions (same cage rack, temperature, humidity, light–dark cycle), except that in the first set of experiments, DCM+S mice received a nutritionally enhanced diet (11.4% fat; 0.4% sodium) while DCM-S mice received the comparable maintenance, non-enhanced diet to foster breeding (5.8% fat; 0.3% sodium). Survival was assessed blindly by animal care technical staff and sexual activity was indirectly verified by successful generation of mouse pups throughout male DCM+S lifespan and directly confirmed by video monitoring (Figure S1D–F). Survival is shown for mice in all different conditions (Figure 1B) and for informative pairwise comparisons (Figure 1C–H). Consistent with our initial non-randomized observations (Figure S1A), the survival of DCM+S mice was significantly prolonged (around 25%, *p* < 0.0001, Figure 1C) when compared to DCM-S mice.

To analyze whether the alternative diet given to DCM+S mice contributed to their enhanced survival, DCM-S littermates were randomly assigned to the same “breeding” diet. There was no significant difference in survival between DCM-S, ND vs. DCM-S, BD, (Figure 1D), while survival of DCM-S, BD mice was significantly shorter when compared to DCM+S, BD mice (*p* < 0.0001, Figure 1E) indicating that “breeding” diet was not a significant factor.

We considered whether other factors besides access to sex may affect the survival of DCM mice. A major difference between DCM+S and DCM-S was the presence of mouse pups. Therefore, we compared the survival of DCM+S mice in cages in which pups after birth were randomly retained for 30 days after birth or removed. Survival was comparable between the groups (median survival—172 vs. 167 days, Figure 1F) suggesting that the presence of pups did not significantly affect outcomes.

To determine if the poor survival of DCM-S mice was due to social interaction, touch or access to sexual activity, a divider with tactile and sensory “social holes” (thirty 0.25 inch holes per divider, Figure S1B) was placed in cages, which permitted the co-housed mice to see, smell and touch each other, but deprived them of sexual activity. Mice that were co-housed with females but were deprived of sexual activity (DCM-S) by this divider had significantly reduced survival by comparison to DCM+S mice that were co-housed without a divider (*p* < 0.01, Figure 1G). Pups were not present in either group.

To ascertain whether continued access to sexual activity was important for survival, we removed access to sexual interaction at an age (13 weeks) when mice have already developed Stage C HF [19,21]. DCM+S mice that were deprived of continuous access to sexual activity starting at 13 weeks, by removal to non-breeding cages, showed a similar survival pattern to DCM-S mice not exposed to sexual activity (Figure 1H). We were unable to assess the effects of sexual activity on females in these experiments since pregnancy itself increases cardiovascular demand and is fatal in mice with HF.

### 2.2. Sexual Interaction Reduces Pleural Effusion and Edema Development

**Figure 2:**
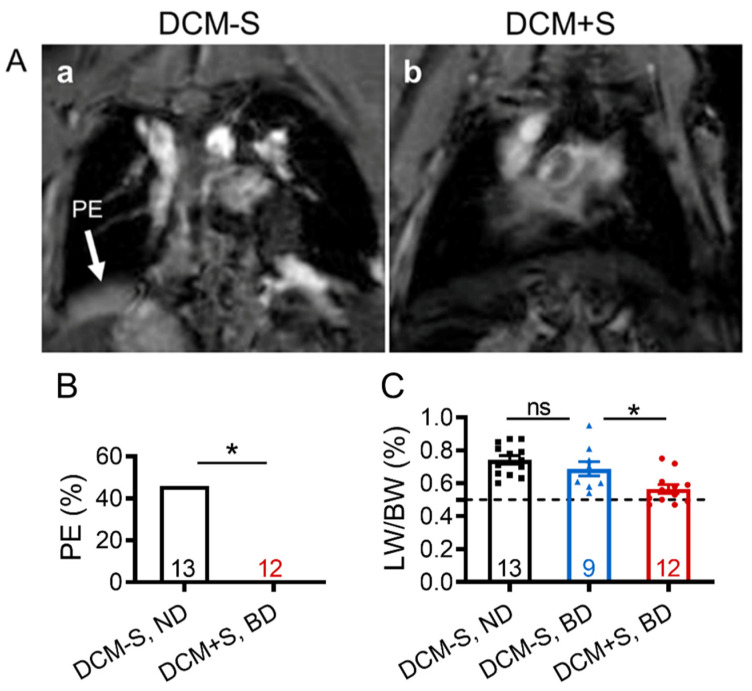
Sexual interaction reduces pleural effusion and edema development in male mice with dilated cardiomyopathy. (**A**) Representative cardiac magnetic resonance imaging (MRI) images of DCM mice without access to sexual interactions (DCM-S (132 days) vs. DCM+S (148 days): presence of pleural effusion (PE, white arrow, panel a) in DCM-S compared to the normal lung pattern (black) in DCM+S (panel b). (**B**) Pleural effusions (PE) prevalence; bars represent percent affected mice. PE was not detected in any control mice without DCM. (**C**) Lung weight to body weight ratio (LW/BW). (**B**,**C**) Groups were male dilated cardiomyopathy (DCM) mice with sexual activity (DCM+S) vs. without sexual activity (DCM-S) on normal (ND) or breeding diet (BD) as measured at 20 weeks of age (Stage D heart failure). The number of DCM mice per group is shown and the value for control mice without DCM is indicated by dashed line (*n* = 7–13). Data are mean ± standard error of the mean (SEM); * *p* < 0.05, ns = non-significant.

Since giving DCM male mice access to sexual interactions with females enhanced survival, we examined whether it may also delay the progression of DCM to HF characterized by edema and pleural effusion. Representative magnetic resonance imaging (MRI) revealed severe pulmonary congestion/pleural effusion in DCM-S (at 132 days) but not in DCM+S (at 148 days) (Figure 2A and Video Figure S2A,B). Necropsy analysis, performed at 20 weeks of age, confirmed that sexual interaction was associated with reduced development of pleural effusion (*p* = 0.015; Figure 2B). The development of edema was significantly higher in DCM-S mice vs. DCM+S mice, measured at 20 weeks of age by lung water or lung weight to body weight ratio (LW/BW) [21], regardless of diet (Figure 2C).

### 2.3. Sexual Interaction Improves Myocardial Contractile Function

**Figure 3:**
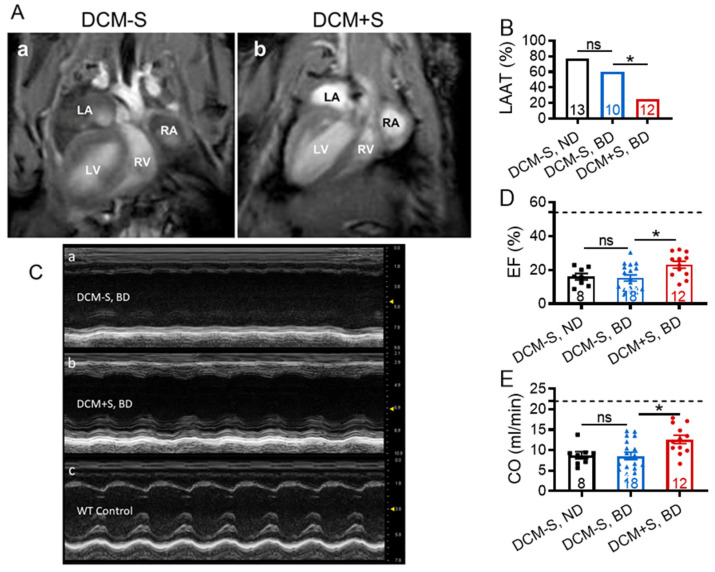
Sexual interaction is associated with improved myocardial contractile function in male mice with dilated cardiomyopathy. (**A**) Representative cardiac magnetic resonance imaging (MRI) images of DCM-S (132 days) versus DCM+S (148 days) showing the presence of ventricular and atrial dilation in DCM-S (panel a) versus DCM+S (panel b). Left atrium (LA) with thrombus; right atrium (RA); left ventricle (LV); right ventricle (RV). White contrast indicates blood flow. (**B**) Percentage of mice with left atrial appendage thrombus (LAAT). LAAT was not detected in any control mice without DCM. (**C**) Representative two-dimensional-guided LV short-axis M-mode images (mm scale on the right y-axis). (**D**) Ejection fraction (EF). (**E**) Cardiac output (CO). (**B**–**E**) Groups were male dilated cardiomyopathy (DCM) mice with sexual activity (DCM+S) vs. without sexual activity (DCM-S) on a normal (ND) or breeding diet (BD) as measured at 20 weeks of age, corresponding to Stage D heart failure. The number of DCM mice per group is shown and the values for control mice without DCM are indicated by dashed lines (*n* = 7–13). Data are mean ± standard error of the mean (SEM); * *p* < 0.05, ns = non-significant.

Representative MRI showed left-ventricular dilation and LAAT formation in DCM-S (at 132 days), which were not evident in DCM+S (at 148 days) (Figure 3A and Video Figure S2A,B). Necropsy analysis, performed at 20 weeks of age, confirmed that sexual deprivation was associated with significant increase in prevalence of LAAT (*p* < 0.05; Figure 3B). Myocardial contractile function or systolic function were measured at 20 weeks of age by transthoracic echocardiography (Table 1). Representative M-mode images of individual mice from DCM-S and DCM+S groups showed differences in morphology, specifically myocardial wall thickness and chamber dimension, and the contractile function of the left ventricle (LV, Figure 3C). The ejection fraction and fractional shortening of the LV, was significantly higher in DCM+S vs. DCM-S groups, representing 46% improvement (Figure 3D). Similarly, the estimated volume of blood pumped per minute (cardiac output, ml/min) was significantly higher in DCM+S vs. DCM-S (Figure 3E).

### 2.4. Sexual Activity Attenuates Heart Failure through Differentiall Activation of Cardiac Contractile Transcription Pathways

Transcriptional networks orchestrate complex physiological and pathological processes. In HF, there is reprogramming of cardiac gene expression with induction of embryonic and contractile protein genes pro-ANP and Myh7 (β-myosin heavy chain) and changes in the relative proportion of Myh7 and Myh6 (α-myosin heavy chain) in the LV. Shift in myosin heavy chain gene expression from the Myh6 to Myh7 isoforms is a hallmark of HF both in mice and human [13,25,26,27,28].

**Figure 4:**
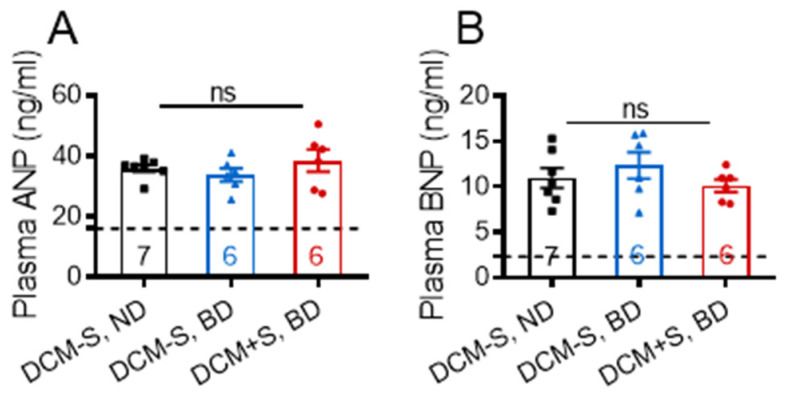
Deprivation of sexual activity does not significantly affect atrial and B-type natriuretic peptide (ANP and BNP) levels in mice with dilated cardiomyopathy (DCM). (**A**) Plasma ANP and (**B**) BNP levels in DCM+S and DCM-S mice. DCM-S, ND (DCM non-breeders, normal diet, black); DCM-S, BD (DCM non-breeders, breeding diet, blue); DCM+S, BD (DCM breeders, breeding diet, red). Dashed line control is shown for mice without DCM, *n* = 7. Data are mean ± standard error of the mean (SEM); ns = non-significant.

To gain insights into the mechanism(s) responsible for the beneficial effects of sexual activity on critical physiological responses such as improvement of heart function and reduction of HF-associated water retention, a comparative microarray analysis of LV gene expression in DCM+S and DCM-S littermates both on BD at 20 weeks of age were performed. The microarray analysis identified only 30 differentially expressed genes (fold-change greater than 1.5; *p* < 0.05 and false discovery rate correction, FDR < 0.05; Figure S3). The bioinformatics analysis revealed DCM as one of mostly attenuated pathologies and Myh7 as the only differentially expressed annotated gene. Consistent with plasma levels (Figure 4A,B), gene levels of pro-ANP (1.00-fold, not significant) and pro-BNP (1.03-fold, not significant) were similarly elevated in DCM+S and DCM-S groups. Expression of other pro-ANP/pro-BNP related genes (transcription factors, processing enzymes and receptors) were not significantly altered (Figure S3).

**Figure 5:**
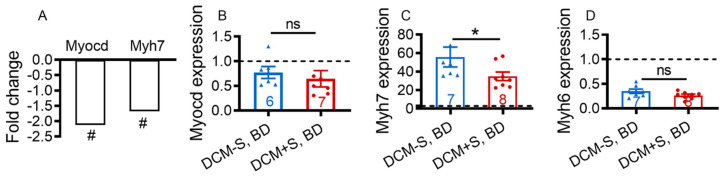
Access to sexual activity differentially activates cardiac contractile transcription pathways in male mice with dilated cardiomyopathy. (**A**) Myocardin (Myocd) and myosin heavy chain 7 (Myh7) fold change (reduction) in left ventricular tissue of DCM+S, BD vs. DCM-S, BD (*n* = 4 littermates/group), determined by microarray. # indicates that differences in gene expression were statistically significant (*p* < 0.05, false discovery rate correction, FDR ≤ 0.05). (B to D) Transcript levels of Myocd (**B**), Myh7 (**C**) and myosin heavy chain 6 (Myh6) (**D**), in DCM-S versus DCM+S, determined by quantitative real-time polymerase chain reaction (PCR). Groups were male dilated cardiomyopathy (DCM) mice with sexual activity (DCM+S) vs. without sexual activity (DCM-S) on normal (ND) or breeding diet (BD) as measured at 20 weeks of age (Stage D heart failure). The number of DCM mice per group is shown and the values for control mice without DCM are indicated by dashed lines (*n* = 7). Data are mean ± standard error of the mean (SEM); *p < 0.05, ns = non-significant.

We focused on DCM-associated differentially expressed annotated gene—Myh7 [25,28] and its upstream transcriptional coactivator myocardin (Myocd) [29,30], which are elevated in human HF and, which appear to affect LV function and survival in HF animals [13,25,28,30]. Myocd and Myh7 expression were reduced 2.12-fold (*p* = 0.002, FDR = 0.04) and 1.67-fold (*p* = 0.012, FDR = 0.05) respectively (Figure 5A), while the Myh6 level was not changed (1.05-fold, not significant) in DCM+S vs. DCM-S. LV expression of other Myocd-regulated genes [30] that encode cardiac cytoskeletal and myofibrillar structural proteins, such as cardiac α-actin-1 (ACTC gene), α-actinin-2 (ACTN2 gene), desmin 18 (DES gene), dystrophin (DMD gene), and tropomyosin-1 (tropomyosin-α chain; TPM1) were not significantly modulated (Figure S3). Reduction of Myh7 and Myocd (not significant trend) transcripts in LV of DCM+S mice was confirmed by quantitative real-time polymerase chain reaction (qRT-PCR) analysis (Figure 5B–D). Down-regulation in relative proportion of Myh7/Myh6 in LV is consistent with improved impaired myocardial contractile function, attenuated HF and prolonged survival in DCM+S vs. DCM-S mice and in accordance with the functional and biomarker role of Myh7 in DCM-HF progression.

### 2.5. Sexual Activity and Lowered Testosterone, Attenuate HF and Prolong Survival

**Figure 6:**
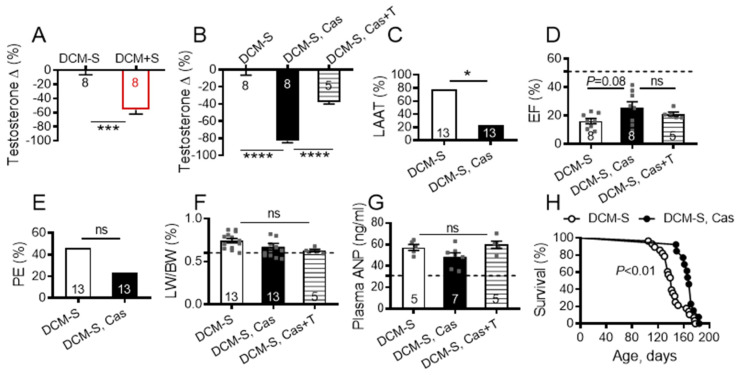
Lowering testosterone improves myocardial contractile function, decreases prevalence of left atrial appendage thrombus (LAAT) and pleural effusion, and prolongs survival in male dilated cardiomyopathy (DCM) mice. (**A**) Plasma testosterone levels (% of non-breeding control mice) in DCM breeders (DCM+S) and non-breeding (DCM-S) littermates. **(B)** Plasma testosterone levels (% of non-breeding control DCM mice) in the castrated (DCM-S, Cas), castrated supplemented with testosterone (DCM-S, Cas+T) and control DCM-S experimental groups. (**C**) Prevalence of LAAT; bars represent percent affected mice. (**D**) Ejection fraction (EF). (**E**) Pleural effusions (PE) prevalence; bars represent percent affected mice. (**F**) Lung weight to body weight ratio (LW/BW). (**G**) Plasma ANP levels. (A–G) Mice analyzed at 20 weeks of age. DCM mice per group is shown; control mice without DCM (dashed line), *n* = 9–10. Data are mean ± standard error of the mean (SEM). * *p* < 0.05, *** *p* < 0.001, **** *p* < 0.0001, ns = not significant. PE and LAAT were not detected in any control mice without DCM. (**H**) Kaplan–Meier survival curve for DCM-S (*n* = 28) vs. DCM-S, Cas mice (*n* = 13). Survival of control mice without DCM were normal and consistent with wild-type C57BL6/J mice.

Married men with or without children have lower testosterone levels than single or unmarried men [31,32] and the biological reasons for these differences are unknown. A limitation of the current study is that it does not define the mechanisms through which access to sexual activity may affect testosterone levels. Consistent with human data, plasma testosterone levels were lower in DCM+S mice than in DCM-S mice, irrespective of diet (Figure 6A), but transcripts for cardiac LV androgen receptor were not significantly altered (1.06-fold, not significant) by sexual interactions. Although a causal effect of endogenous and exogenous testosterone on heart function is controversial and not fully understood [33,34,35], some animal and clinical studies suggest that testosterone may negatively affect cardiac contractile function [36,37,38,39,40,41,42] and cause salt and water retention, contributing to risk of edema [33].

To determine whether lower plasma testosterone levels may contribute to the enhanced survival in DCM+S mice, we examined the effect of castration (at 4 weeks) on outcomes for DCM-S mice at 20 weeks of age. Castration significantly reduced plasma testosterone levels by comparison to non-castrated mice (Figure 6B), but did not significantly alter cardiac LV androgen receptor transcript levels (1.16-fold, not significant). There were significant decreases in the prevalence of LAAT formation (*p* < 0.05: Figure 6C) and non-significant trends towards better cardiac contractility (Figure 6D), reduced pleural effusions (Figure 6E) and possibly edema (LW/BW%, Figure 6F) in the castrated group as compared to non-castrated DCM-S controls. Plasma testosterone levels were partially restored in the castrated DCM-S mice with subcutaneously implanted exogenous testosterone-containing pellets (Figure 6B). Restoration of plasma testosterone levels was associated with a non-significant trend toward impaired cardiac contractility (Figure 6D), but did not change edema (LW/BW%, Figure 6F). There was no difference between the groups in plasma (Figure 6G) and LV-myocardium mRNA ANP levels (1.00 fold; not significant). Castration significantly decreased Myocd mRNA (1.58-fold; *p* = 0.023, FDR = 0.039) but not Myh7 (1.18-fold; not significant) and Myh6 (1.01-fold; not significant) transcripts in LV-myocardium vs. non-castrated DCM-S controls. Future experimental studies should explore the direct causative effects of precise doses of testosterone on beneficial alteration of the Myocd-Myh7 axis in LV as DCM progresses through A to D HF stages.

Castrated DCM-S had significantly improved survival when compared with non-castrated DCM-S control mice (Figure 5G). Associations of castration with prolonged survival have been reported in other cardiovascular mouse models [36,37,43,44]. Castration has been associated with prolonged male survival in humans [45,46,47] and many other mammals, including primates, sheep, cats, dogs, rats, and mice [47,48].

In conclusion, sexual deprivation in male mice with DCM led to accelerated declines in heart function, progressive fluid retention, higher testosterone levels and shorter lives. The fundamental question how sexual activity reduces testosterone levels remains to be answered and the underlining molecular mechanisms remain to be discovered. Since these experiments were performed in an experimental model of DCM in male mice, there are important potential limitations to the generalizability of our findings. Despite these shortcomings, these findings suggest that sexual interactions may have beneficial effects in DCM with HF, which may be mediated in part through their effects on testosterone. As such, these data suggest a conceptual paradigm that in HF, sexual interactions may alter cardiac transcription networks to reduce HF progression and improve survival.

## 3. Materials and Methods

### 3.1. Animals

All experimental activities were reviewed and approved by the Institutional Animal Care and Use Committee at the University of Tennessee Health Science Center (15–050 and 17–059) and/or at the University of Arizona College of Medicine—Phoenix (17–303), and conducted within AAALACi accredited facilities in accordance with the National Institutes of Health (NIH) Guide for the Care and Use of Laboratory Animals. All mice were housed on the same rack system (Optimice—Animal Care Systems, Centennial, CO, USA) in an AAALACi accredited facility with 12:12 light cycle in accordance to the environmental parameters outlined in the 8th edition of the Guide for the Care and Use of Laboratory Animals. Mice were fed a standard/normal diet (ND; Envigo Teklad 7912, Madison, WI, USA) or breeder diet (BD; Teklad 7904) and provided either hyperchlorinated water via facility automated watering lixits or autoclaved water bottles ad lib throughout the study.

Male and female (only experimentally used for socialization/breeding) C57BL/6J littermate mice with or without DCM genetic phenotype were used. DCM mice develop progressive HF, in the setting of preserved kidney function, through the action of a transgene containing the CREB_S133A_ transcription factor that is targeted to cardiomyocytes via the alpha myosin heavy chain promoter [13,16,17,18,19,20,21]. Mice were randomly assigned to groups and housed in one of five lifestyle housing configurations: (1) male DCM + two female wild-type (WT) breeders in one box (DCM+S with access to sexual activity, BD); (2) male DCM cohoused with additional 2–4 male littermates in one box (DCM-S, ND); (3) male DCM cohoused with additional 2–4 males in one box (DCM-S, BD); (4) male DCM housed in boxes with commercially available longitudinal tactile and sensory social hole divider (hole diameter = 0.25” and there are 30/each divider; Unimice Cage Divider Kit w/Social Holes, Animal Care Systems, Centennial, CO) + two WT females on opposite side (DCM+S without access to sexual activity, BD); (5) A subset of male DCM+S mice, breeder diet was shifted to non-breeding cages at 13 weeks of age. A specific number of mice in each subgroup were randomly designated for survival studies as indicated (Figure 1B–F; Figure 3D). The remaining mice in each subgroup were euthanized at 20 weeks of age by an overdose of isoflurane for blood and tissue collection. Blood was collected via cardiocentesis in prepared ethylenediaminetetraacetic acid (EDTA)-aprotinin syringes to block coagulation and proteolysis of targeted proteins. Blood samples were centrifuged at 3000 rpm for 20 min at 4 °C, the resulting plasma was aliquoted, and stored at −80 °C until analysis. Sexual activity was directly monitored using camera array video recording system for mouse observation as described [49] and, indirectly, recording generation of mouse pups throughout male DCM+S lifespan. Animal care staff and lab members monitored the colony daily for health and behavioral changes for the following endpoint criteria: weight loss >20%, increased respiratory rate/effort, and hunched posture were used to prevent suffering. All analysis and health/death reports (mice found dead prior to developing any endpoint criteria) were recorded by investigators and animal facility technicians blinded to mouse genotype.

### 3.2. Echocardiography

Transthoracic images in two-dimensional and M-mode parasternal long-axis and short-axis acoustic windows were performed by an echocardiographer blinded to mouse genotype using a Vevo 2100 Imaging System (Visual Sonic Inc., Toronto, Canada) as we have described previously [16,17,19,21]. The ejection fraction (EF, %) from eight of 41 mice has been reported [21]. Fur from the ventral thorax was removed by chemical depilatory cream (Nair, Church & Dwight Co., Inc., Ewing, NJ, USA) one day before the echocardiographic studies. Mice were anesthetized with 1.5% inhaled isoflurane in oxygen. All measurements were recorded under the same physiological conditions: 450 ± 50 bpm and rectal body temperature 37 ± 0.5 °C. There was no significant difference in age and body weight at the time of imaging between all mice (Table 1). The short-axis M-mode recordings were analyzed using Vevo LAB^®^ (version 3.1.0) software (Visual Sonic Inc., Toronto, Canada); LV trace was used to outline three complete cardiac cycles and results averaged for each mouse. The fractional shortening (FS, %), EF, %, cardiac output (CO, mL/min), were calculated according to VisualSonics standard equations.

### 3.3. Magnetic Resonance Imaging (MRI)

All MRI images were acquired using 7.0T small animal, 30-cm horizontal-bore magnet with Biospec Avance III spectrometer (Bruker, Billerica, MA, USA) with a high-power 116-mm gradient insert (60 G/m) at the Barrow-ASU Center for Preclinical Imaging (Phoenix, AZ, USA). Mouse anesthesia was induced and maintained using 1–2% isoflurane in medical air. Respiratory and ECG signals were monitored and used for gating throughout the image acquisitions. Body temperature was supported by a recirculating water heater, set to 37 °C. Mice were positioned prone within the animal bed/cradle using three-point fixation. During each imaging session, a series of transverse, sagittal, and coronal scout images through the heart [FLASH sequence, TR/TE = 50/2.7 ms, field of view (FOV) = 3 × 3 cm, matrix = 128 × 128, slice thickness = 0.5 mm, number of averages = 5]. Bright-blood long-axis cardiac images acquired [IntraGate FLASH (Bruker, Billerica, MA, USA), TR = 8 ms, TE = 3 ms, number of repetitions = 300, 10 cardiac frames, 256 × 256 matrix, FOV = 35 x 35 mm, 6 slices] oriented through the left atrium. MRI data were analyzed using ImageJ (1.51j8, NIH, Bethesda, MD, USA) on a lab workstation.

### 3.4. Left Atrial Appendage Thrombus, Lung Edema and Pleural Effusion Analysis

Left atrial appendage thrombus (LAAT) formation was assessed by necropsy analysis of mouse hearts. Pleural effusions were assessed by necropsy analysis of the mouse thoracic cavity and quantified as a percent of its prevalence in the group. Lung edema was assessed by the lung weight-to-body weight ratio (LW/BW, %). The LW/BW was calculated as right lung plus left lung wet weight divided by body weight as described previously [16,17,19,21].

### 3.5. Surgical Castration

A subset of DCM males at 4 weeks of age underwent standard surgical castration. Anesthesia was induced with 3–5% isoflurane in oxygen and maintained at 1.0–2.0% isoflurane in oxygen via a nose cone. Pre-operative buprenorphine (0.1 mg/kg SC) was administered for analgesia. The surgical site was aseptically prepared and the mouse was maintained on a heated surgical platform in dorsal recumbency. A 5 mm incision was made with a #15 scalpel blade in the ventral midline of the scrotum and the underlying tunica. Using digital pressure one testicle was exposed from the incision and further removed with caudal traction from the opening. A hot bead (Germinator 500, Electron Microscopy Sciences, Hatfield, PA, USA) heated hemostat was clamped across the cord to cauterize and the testicle was removed by tearing to aid with hemostasis. The second testicle was excised through the same incision and the procedures repeated. After confirming hemostasis of the cords, the tissue was allowed to retract into the scrotum and the incision closed with surgical glue (3M Vetbond, St. Paul, MN, USA). Mice were recovered in a box lined with paper towels on a circulating water heating pad. Once fully conscious and ambulatory, mice were returned to their home enclosures.

### 3.6. Testosterone Pellet Implantation

For testosterone replacement study, while still under anesthesia from the castration, a subset of mice received a subcutaneous slow release testosterone pellet (5.0 mg/pellet; 90-day release; Innovative Research of America, Sarasota, FL, USA). The fur was shaved in a 2 × 2 cm square and skin was aseptically prepared between the shoulder blades. A 5 mm incision was made in the skin layer and mild blunt dissection with scissors of the subcutaneous space was performed prior to pellet insertion. The skin was closed using surgical glue (3M Vetbond, St. Paul, MN, USA).

### 3.7. RNA Isolation and Microarray Analysis

Gene expression differences were analyzed on DCM background, between breeder (*n* = 4) and non-breeder (*n* = 4) male littermates fed with a breeder diet (Teklad 7904) at 20 weeks of age. Hearts were excised, snap frozen in liquid nitrogen, stored at −80 °C until RNA isolation. Total RNA samples were isolated from the left ventricular tissue by using RNeasy Mini Kit (Qiagen, Hilden, Germany) and treated with DNase I (Invitrogen, Carlsbad, CA, USA). For quality control and purity, RNA samples were run on Agilent Nano Chip by Molecular Resource Center, University of Tennessee Health Science Center (Memphis, TN, USA).

The microarray study was performed in the Molecular Resource Center, UTHSC (Memphis, TN, USA). DNase treated total RNA amounting 200 ng was amplified, fragmented and labeled using Genechip WT Plus Reagent Kit (Thermo Fisher Scientific, Waltham, MA, USA) according to Affymetrix Protocols. Samples were hybridized to Affymetrix Clariom S arrays (Thermo Fisher Scientific, Waltham, MA, USA), washed, stained and scanned according to Affymetrix protocols. Arrays were scanned on a GeneChip Scanner 3000 7G (Thermo Fisher Scientific). Once the arrays were scanned, the data were log_2_ transformed and normalized using the Affymetrix expression console. The normalized data matrix was loaded into R to gather statistics and determine differential expression. The mean, variance, standard deviation, and standard error of the mean were calculated for each gene across each condition. Pearson’s correlation coefficient was graphed in order to identify sample outliers in each condition. At that time no outliers were determined. A principal component analysis was performed to determine different clusters in the samples. The fold-change was then calculated for all genes. Welch’s *t* test was implemented in order to determine significance for each gene. The *p* values were then adjusted for multiplicity using the Benjamini–Hochberg method. Only genes with fold-change greater than 1.5 (upregulated or downregulated), *p* value < 0.05 and an adjusted *p* value, and an FDR (false discovery rate correction) < 0.05 were considered differentially expressed. The expression of the CREB gene was not different between the groups (fold-change = 1.05, *p* = 0.78). iPathway Guide analysis was used to identify affected pathologies/disease and the differentially expressed genes associated with identified pathology–annotated genes. iPathwas Guide scored diseases using the Kyoto Encyclopedia of Genes and Genomes (KEGG, Kanehisa Laboratories, Japan) database (Release 81.0 + /0120, Jan 17). For each disease, the number of differentially expressed genes annotated to a disease term is compared to the number of genes expected just by chance.

### 3.8. Quantitative Real-Time Polymerase Chain Reaction (qRT-PCR)

qRT-PCR reactions were performed in triplicate as described previously [16,17,19,21]. Total RNA was extracted from snap-frozen heart tissue using the RNeasy^®^ Mini Kit (Qiagen, Germantown, MD, USA). First strand cDNA synthesis was performed with 1 μg of total RNA (Transcriptor First Strand cDNA Synthesis Kit, Roche, Basel, Switzerland). qRT-PCR was performed using the LightCycler^®^ 480 System (Roche Diagnostics Ltd., Switzerland), following the manufacturer’s protocol. Specific primers were: tgctcagagctcaagaaggat and cccagccatctcctctgtt for Myh6 (NM_001164171.1), ctcagagctcaagcgggata and ccagccatctcctctgtca for Myh7 (NM_080728.2), gcaagggcagaaacaggtc and atctgagcagttggaatggac for Myocd (NM_145136.4). qRT-PCR was performed at: 95 °C for 5 min, followed by 40 cycles of 95 °C (10 s), 60 °C (30 s), and 72 °C (10 s). Samples were normalized to *Polr2a* (DNA-directed RNA polymerase II subunit RPB1) as an internal control.

### 3.9. Enzyme Immunoassay

Plasma ANP (as N terminus-ANP), BNP (as C terminus-BNP) and testosterone were measured by enzyme immunoassays according to the manufacturer’s protocols (Phoenix Pharmaceuticals, Inc., Burlingame, CA, USA; Enzo Life Science Inc., Farmingdale, NY, USA).

### 3.10. Statistical Analysis

Statistical analysis was performed with Graph Pad Prism 7.0 software (GraphPad Software, La Jolla, CA, USA). Survival was analyzed by the Kaplan–Meier method and the comparison of two groups was assessed by the Mantel–Cox test. Differences among more than two groups were analyzed by one-way analysis of variance (ANOVA) using pairwise Tukey’s post-hoc correction. Differences between two groups were analyzed by a non-parametric Mann–Whitney t test. Categorical data (pleural effusions, LAAT) were analyzed by Fisher’s exact test. Differences were considered to be significant if the two-tailed *p* ≤ 0.05. The number of animals (n) is indicated in the figures or legends. Data were expressed as mean ± SEM.

## 4. Conclusions

Heart failure (HF) causes death and disability in millions of people worldwide. Research has focused on medical therapy and the impact of lifestyle on outcomes remains poorly understood. Male HF patients generally indicate that sexual activity enhances their quality of life, but they are often fearful because the safety and effects of sexual activity on HF are not fully known. We show that sex is not only safe, but beneficial in experimental HF. It alters physiology and gene expression to slow the progression of heart dysfunction, delay the onset of HF as assessed by edema development and prolong life. These data suggest a new conceptual paradigm for HF which recognizes that lifestyle and behavioral activities may modify physiology, gene expression and the course of disease.

## Figures and Tables

**Table 1:** Physiological parameters and echocardiographic comparisons of all experimental groups.

Group:	WT, ND	DCM-S, ND	DCM-S, BD	DCM+S, BD	^1^*p*-Value
Group Size (n):	13	8	18	12	
Age (days):	137 ± 1	139 ± 1	139 ± 0.5	137 ± 1	0.31
Body Weight (g):	29 ± 0.7	29 ± 0.6	29 ± 0.5	30 ± 0.6	0.20
Heart Rate (bpm):	479 ± 12	420 ± 21	440 ± 22	480 ± 20	0.11
Ejection Fraction (%):	54 ± 2	16 ± 2	15 ± 2	23 ± 2	0.0001
Fractional Shortening (%):	28 ± 1	7 ± 1	7 ± 1	11 ± 1	0.0001
Cardiac Output (mL/min):	22 ± 1	9 ± 1	9 ± 1	13 ± 1	0.0001

^1^ Data reported as mean ± standard error of the mean (SEM). Abbreviations: wild-type (WT) control mice without dilated cardiomyopathy (DCM); normal diet (ND); DCM mice without access to sexual interactions (DCM-S); DCM mice with access to sexual interactions (DCM+S); breeding diet (BD).

## Supplementary Materials

The following are available online at https://www.mdpi.com/1422-0067/21/15/5450/s1. Figure S1. Deprivation of sexual activity shortens survival in mice with DCM. Figure S2. Sexual deprivation affects cardiac function in DCM male mice. Figure S3. Sexual activity deprivation modulates expression of contractile protein genes Myocd and Myh7 levels in mice with DCM. Fold-change in transcript levels of the differentially expressed genes analyzed by microarray in left ventricular (LV) tissue of DCM+S vs. DCM-S, on breeding diet (*n* = 4 per group).

## Abbreviations

ACTC1	α-actin-1
ACTC2	α-actinin-2
ANP	atrial natriuretic peptide
BD	breeding diet
BNP	b-type natriuretic peptide
CO	cardiac output
Cas	castrated
Cas+T	castrated with supplemented testosterone
DES	desmin
DVR	digital video recorders
DCM	dilated cardiomyopathy
DCM+S	dilated cardiomyopathy with access to sexual activity
DCM-S	dilated cardiomyopathy without access to sexual activity
DMD	dystrophin
EF	ejection fraction
ELISA	enzyme-linked immunosorbent assay
FDR	false discovery rate
FC	fold-change
FS	fractional shortening
HF	heart failure
LAAT	left atrial appendage thrombus
LA	left atrium
LV	left ventricle
LW/BW	lung weight-to-body weight ratio
MRI	magnetic resonance imaging
Myocd	myocardin
Myh6	myosin heavy chain 6
Myh7	myosin heavy chain 7
NP	natriuretic peptide
ND	normal diet
PE	pleural effusion
qRT-PCR	quantitative real-time polymerase chain reaction
RA	right atrium
RV	right ventricle
T	testosterone
TPM1	tropomyosin-1

## References

[1] Jessup M., Brozena S. (2003). Heart failure. N. Engl. J. Med..

[2] Metra M., Teerlink J.R. (2017). Heart failure. Lancet.

[3] Weintraub R.G., Semsarian C., Macdonald P. (2017). Dilated cardiomyopathy. Lancet.

[4] Lindau S.T., Schumm L.P., Laumann E.O., Levinson W., O’Muircheartaigh C.A., Waite L.J. (2007). A Study of Sexuality and Health among Older Adults in the United States. N. Engl. J. Med..

[5] Levine G.N., Steinke E.E., Bakaeen F.G., Bozkurt B., Cheitlin M.D., Conti J.B., Foster E., Jaarsma T., Kloner R.A., Lange R.A. (2012). Sexual Activity and Cardiovascular Disease: A Scientific Statement From the American Heart Association. Circulation.

[6] Baert A., Pardaens S., De Smedt D., Puddu P.E., Ciancarelli M.C., Dawodu A., De Sutter J., De Bacquer D., Clays E. (2019). Sexual Activity in Heart Failure Patients: Information Needs and Association with Health-Related Quality of Life. Int. J. Environ. Res. Public Heal..

[7] Mandras S., Uber P.A., Mehra M.R. (2007). Sexual Activity and Chronic Heart Failure. Mayo Clin. Proc..

[8] Jaarsma T. (2017). Reply to ‘Sexual function of patients with heart failure: Distinct phenotypes distinct sexual function?’ by Konstantinos Koutsampasopoulos. ESC Hear. Fail..

[9] Jaarsma T., Fridlund B., Mårtensson J. (2014). Sexual Dysfunction in Heart Failure Patients. Curr. Hear. Fail. Rep..

[10] Kolbe N., Kugler C., Schnepp W., Jaarsma T. (2016). Sexual Counseling in Patients with Heart Failure. J. Cardiovasc. Nurs..

[11] Fentzke R.C., Korcarz C.E., Lang R.M., Lin H., Leiden J.M. (1998). Dilated cardiomyopathy in transgenic mice expressing a dominant-negative CREB transcription factor in the heart. J. Clin. Investig..

[12] Spencer K.T., Collins K., Korcarz C.E., Fentzke R., Lang R.M., Leiden J.M. (2000). Effects of exercise training on LV performance and mortality in a murine model of dilated cardiomyopathy. Am. J. Physiol. Circ. Physiol..

[13] Watson P.A., Birdsey N., Huggins G.S., Svensson E., Heppe D., Knaub L. (2010). Cardiac-specific overexpression of dominant-negative CREB leads to increased mortality and mitochondrial dysfunction in female mice. Am. J. Physiol. Circ. Physiol..

[14] Gladysheva I.P., Wang N., McNamee R., Houng A.K., A Mohamad A., Fan T.M., Reed G. (2012). Corin overexpression improves cardiac function, heart failure, and survival in mice with dilated cardiomyopathy. Hypertension.

[15] Wang N., Gladysheva I.P., Fan T.-H.M., Sullivan R., Houng A.K., Reed G. (2013). Atrial natriuretic peptide affects cardiac remodeling, function, heart failure, and survival in a mouse model of dilated cardiomyopathy. Hypertension.

[16] Tripathi R., Wang D., Sullivan R., Fan T.-H.M., Gladysheva I.P., Reed G.L. (2016). Depressed Corin Levels Indicate Early Systolic Dysfunction Before Increases of Atrial Natriuretic Peptide/B-Type Natriuretic Peptide and Heart Failure Development. Hypertension.

[17] Tripathi R., Sullivan R., Fan T.-H.M., Wang D., Sun Y., Reed G.L., Gladysheva I.P. (2017). Enhanced heart failure, mortality and renin activation in female mice with experimental dilated cardiomyopathy. PLoS ONE.

[18] Sullivan R., Mehta R.M., Tripathi R., Gladysheva I.P., Reed G.L. (2019). Normalizing Plasma Renin Activity in Experimental Dilated Cardiomyopathy: Effects on Edema, Cachexia, and Survival. Int. J. Mol. Sci..

[19] Tripathi R., Sullivan R., Fan T.-H.M., Houng A.K., Mehta R.M., Reed G.L., Gladysheva I.P. (2019). Cardiac-Specific Overexpression of Catalytically Inactive Corin Reduces Edema, Contractile Dysfunction, and Death in Mice with Dilated Cardiomyopathy. Int. J. Mol. Sci..

[20] Merlo M., Cannata A., Gobbo M., Stolfo D., Elliott P.M., Sinagra G. (2017). Evolving concepts in dilated cardiomyopathy. Eur. J. Hear. Fail..

[21] Hershberger R.E., Hedges D.J., Morales A. (2013). Dilated cardiomyopathy: The complexity of a diverse genetic architecture. Nat. Rev. Cardiol..

[22] Yancy C.W., Jessup M., Bozkurt B., Butler J., Casey D.E., Jr Drazner M.H., Fonarow G.C., Geraci S.A., Horwich T., Januzzi J.L. (2013). 2013 ACCF/AHA guideline for the management of heart failure: Executive summary: A report of the American College of Cardiology Foundation/American Heart Association Task Force on practice guidelines. Circulation.

[23] Houser S.R., Margulies K.B., Murphy A.M., Spinale F.G., Francis G.S., Prabhu S.D., Rockman H.A., Kass D.A., Molkentin J.D., A Sussman M. (2012). Animal Models of Heart Failure. Circ. Res..

[24] Tallent B.R., Lifshitz J. (June 2018). Creating an Inexpensive Camera Array for Rodent Observation.

[25] Grewal J., Siu S.C., Ross H.J., Mason J., Balint O.H., Sermer M., Colman J.M., Silversides C.K. (2009). Pregnancy Outcomes in Women With Dilated Cardiomyopathy. J. Am. Coll. Cardiol..

[26] Lompre A.-M., Schwartz K., D’Albis A., Lacombe G., Van Thiem N., Swynghedauw B. (1979). Myosin isoenzyme redistribution in chronic heart overload. Nature.

[27] Edwards B.S., Ackermann D.M., Lee M.E., Reeder G.S., Wold L.E., Burnett J.C. (1988). Identification of atrial natriuretic factor within ventricular tissue in hamsters and humans with congestive heart failure. J. Clin. Investig..

[28] Razeghi P., Young M.E., Alcorn J.L., Moravec C.S., Frazier O., Taegtmeyer H. (2001). Metabolic gene expression in fetal and failing human heart. Circulation.

[29] Rajabi M., Kassiotis C., Razeghi P., Taegtmeyer H. (2007). Return to the fetal gene program protects the stressed heart: A strong hypothesis. Hear. Fail. Rev..

[30] Wang D.-Z., Chang P.S., Wang Z., Sutherland L., Richardson J.A., Small E., Krieg P.A., Olson E.N. (2001). Activation of Cardiac Gene Expression by Myocardin, a Transcriptional Cofactor for Serum Response Factor. Cell.

[31] Mikhailov A.T., Torrado M. (2012). In Search of Novel Targets for Heart Disease: Myocardin and Myocardin-Related Transcriptional Cofactors. Biochem. Res. Int..

[32] Holmboe S.A., Priskorn L., Jørgensen N., Skakkebaek N.E., Linneberg A., Juul A., Andersson A.-M. (2017). Influence of marital status on testosterone levels—A ten year follow-up of 1113 men. Psychoneuroendocrinology.

[33] Alvergne A., Faurie C., Raymond M. (2009). Variation in testosterone levels and male reproductive effort: Insight from a polygynous human population. Horm. Behav..

[34] Basaria S., Coviello A.D., Travison T.G., Storer T.W., Farwell W.R., Jette A.M., Eder R., Tennstedt S., Ulloor J., Zhang A. (2010). Adverse Events Associated with Testosterone Administration. N. Engl. J. Med..

[35] Xu L., Freeman G., Cowling B.J., Schooling M. (2013). Testosterone therapy and cardiovascular events among men: A systematic review and meta-analysis of placebo-controlled randomized trials. BMC Med..

[36] Kloner R.A., Carson C., Dobs A., Kopecky S., Mohler E.R. (2016). Testosterone and Cardiovascular Disease. J. Am. Coll. Cardiol..

[37] Cavasin M.A., Sankey S.S., Yu A.-L., Menon S., Yang X.-P. (2003). Estrogen and testosterone have opposing effects on chronic cardiac remodeling and function in mice with myocardial infarction. Am. J. Physiol. Circ. Physiol..

[38] Gao X.M., Agrotis A., Autelitano D.J., Percy E., Woodcock E.A., Jennings G.L., Dart A.M., Du X.J. (2003). Sex hormones and cardiomyopathic phenotype induced by cardiac beta 2-adrenergic receptor overexpression. Endocrinology.

[39] Bocchi E.A., Carvalho V.O., Guimaraes G.V. (2008). Inverse correlation between testosterone and ventricle ejection fraction, hemodynamics and exercise capacity in heart failure patients with erectile dysfunction. Int. Braz. J. Urol..

[40] Baggish A.L., Weiner R.B., Kanayama G., Hudson J.I., Picard M.H., Hutter A.M., Pope H.G. (2010). Long-Term Anabolic-Androgenic Steroid Use Is Associated With Left Ventricular Dysfunction. Circ. Hear. Fail..

[41] Huang C.-K., Lee S.O., Chang E., Pang H., Chang C. (2016). Androgen receptor (AR) in cardiovascular diseases. J. Endocrinol..

[42] Junior R.R., Ronconi K., Jesus I., Almeida P., Forechi L., Vassallo D.V., Guatimosim S., Stefanon I., Fernandes A. (2018). Testosterone deficiency prevents left ventricular contractility dysfunction after myocardial infarction. Mol. Cell. Endocrinol..

[43] Kattih B., Elling L.S., Weiss C., Bea M., Zwadlo C., Bavendiek U., Bauersachs J., Heineke J. (2019). Anti-androgenic therapy with finasteride in patients with chronic heart failure—A retrospective propensity score based analysis. Sci. Rep..

[44] Lin C.-Y., Lin M.-T., Cheng R.-T., Chen S.-H. (2010). Testosterone Depletion by Castration May Protect Mice from Heat-Induced Multiple Organ Damage and Lethality. J. Biomed. Biotechnol..

[45] Zhao G., Moore D.J., Kim J.I., Lee K.M., O’Connor M.R., Duff P.E., Yang M., Lei J., Markmann J.F., Deng S. (2011). Inhibition of Transplantation Tolerance by Immune Senescence Is Reversed by Endocrine Modulation. Sci. Transl. Med..

[46] Hamilton J.B., Mestler G.E. (1969). Mortality and Survival: Comparison of Eunuchs with Intact Men and Women in a Mentally Retarded Population. J. Gerontol..

[47] Min K.-J., Lee C.-K., Park H.-N. (2012). The lifespan of Korean eunuchs. Curr. Boil..

[48] Gems D. (2014). Evolution of sexually dimorphic longevity in humans. Aging.

[49] Brooks R.C., Garratt M.G. (2016). Life history evolution, reproduction, and the origins of sex-dependent aging and longevity. Ann. N. Y. Acad. Sci..

