# Supplementary material for: In Experimental Dilated Cardiomyopathy Heart Failure and Survival Are Adversely Affected by a Lack of Sexual Interactions
Source: Int J Mol Sci. 2020 Jul 30;21(15):5450. doi: 10.3390/ijms21155450 (PMC7432836; doi:10.3390/ijms21155450)
Supplement: Supplementary file 1 [file ijms-21-05450-s001.pdf]

International Journal of
Molecular Sciences

MDPI

Supplementary Materials: Supplementary Materials can be found at www.mdpi.com/xxx/s1.

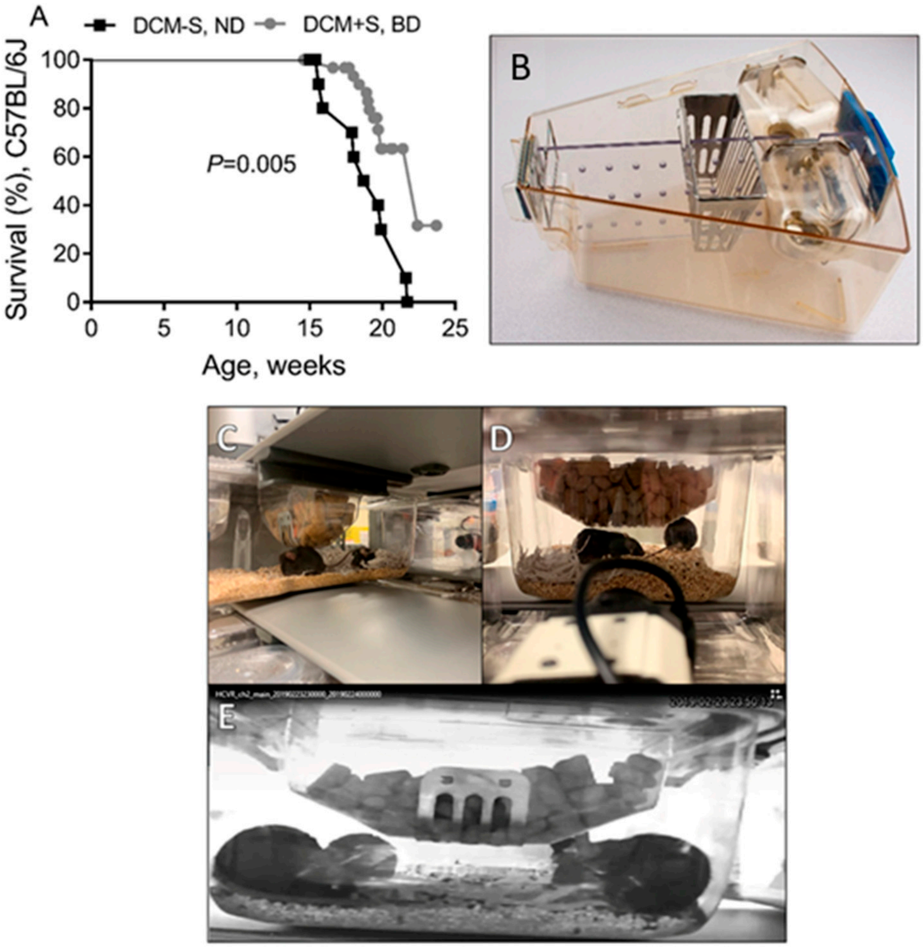

Figure 1. Deprivation of sexual activity shortens survival in mice with DCM. (**A**) Kaplan Meier survival curve of DCM+S (n = 32) vs. DCM-S (n = 19), on C57BL/6J genetic background. (**B**) Example of the porous social divider (Unimice Cage Divider Kit w/Social Holes, Animal Care Systems, Centennial, CO, USA). (**C,D**) Representative camera setup for breeding activity recording. (**E**) Example of a still image from video recording using multi-camera digital video recorders (DVR).

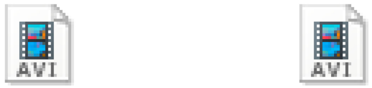

DCM-S, BD.avi DCM+S, BD.avi

Figure 2. Sexual deprivation affects cardiac function in DCM male mice. (**A,B**) Representative cardiac MRI videos of (**A**) DCM-S (132 days) presence of the ventricular and atrial dilation, left atrial thrombosis and pleural effusion compared to (**B**) DCM+S (148 days). Click on the included image to play the video of MRI scans.

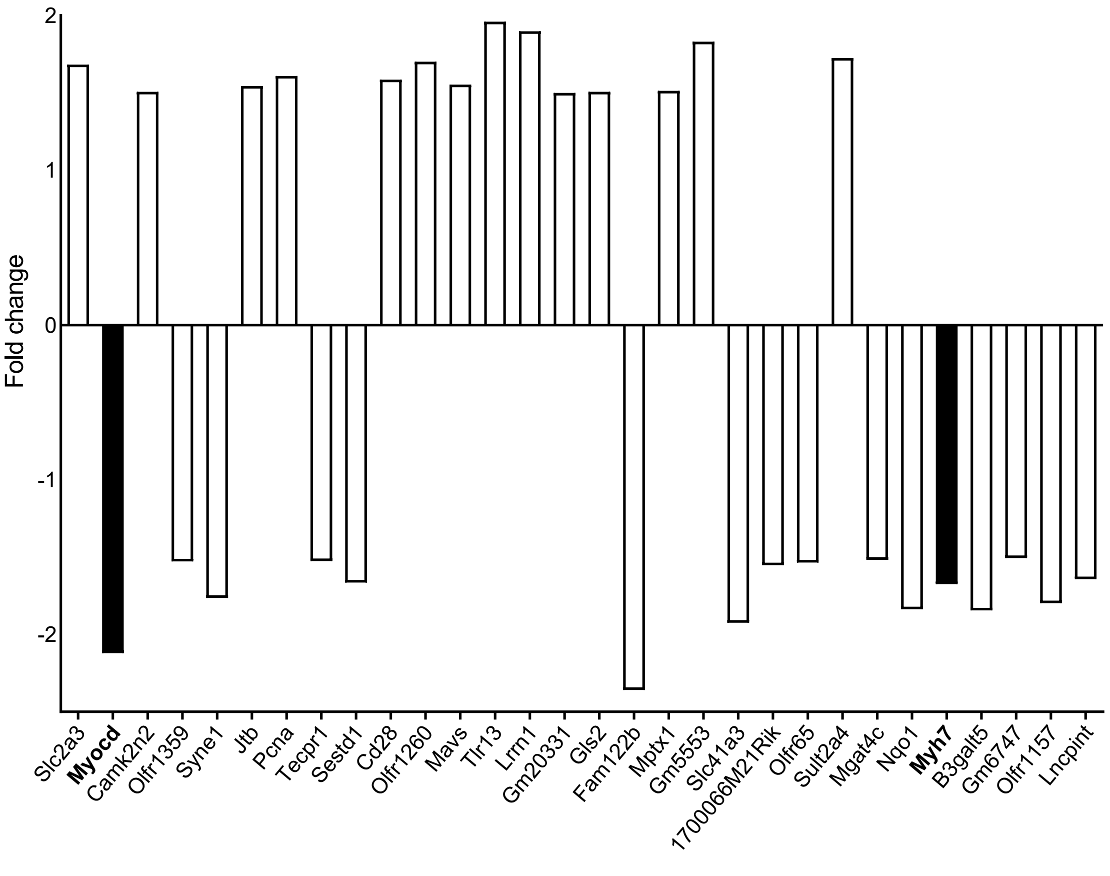

Figure 3. Sexual activity deprivation modulates expression of contractile protein genes Myocd and Myh7 levels in mice with DCM. Fold-change in transcript levels of the differentially expressed genes analyzed by microarray in left ventricular (LV) tissue of DCM+S vs. DCM-S, on breeding diet (n = 4 per group).

© 2020 by the authors. Submitted for possible open access publication under the terms and conditions of the Creative Commons Attribution (CC BY) license (http://creativecommons.org/licenses/by/4.0/).
